# An integrated cytokine and kynurenine network as the basis of neuroimmune communication

**DOI:** 10.3389/fnins.2022.1002004

**Published:** 2022-11-24

**Authors:** Trevor W. Stone, Felix I. L. Clanchy, Yi-Shu Huang, Nien-Yi Chiang, L. Gail Darlington, Richard O. Williams

**Affiliations:** ^1^The Kennedy Institute of Rheumatology, NDORMS, University of Oxford, Oxford, United Kingdom; ^2^Department of Internal Medicine, Ashtead Hospital, Ashtead, United Kingdom

**Keywords:** neuroimmune interface, kynurenine, kynurenic acid, quinolinic acid, cytokines, immune-neural communication, glutamate, homing

## Abstract

Two of the molecular families closely associated with mediating communication between the brain and immune system are cytokines and the kynurenine metabolites of tryptophan. Both groups regulate neuron and glial activity in the central nervous system (CNS) and leukocyte function in the immune system, although neither group alone completely explains neuroimmune function, disease occurrence or severity. This essay suggests that the two families perform complementary functions generating an integrated network. The kynurenine pathway determines overall neuronal excitability and plasticity by modulating glutamate receptors and GPR35 activity across the CNS, and regulates general features of immune cell status, surveillance and tolerance which often involves the Aryl Hydrocarbon Receptor (AHR). Equally, cytokines and chemokines define and regulate specific populations of neurons, glia or immune system leukocytes, generating more specific responses within restricted CNS regions or leukocyte populations. In addition, as there is a much larger variety of these compounds, their homing properties enable the superimposition of dynamic variations of cell activity upon local, spatially limited, cell populations. This would in principle allow the targeting of potential treatments to restricted regions of the CNS. The proposed synergistic interface of ‘tonic’ kynurenine pathway affecting baseline activity and the superimposed ‘phasic’ cytokine system would constitute an integrated network explaining some features of neuroimmune communication. The concept would broaden the scope for the development of new treatments for disorders involving both the CNS and immune systems, with safer and more effective agents targeted to specific CNS regions.

## Introduction

Many neuropsychiatric disorders are accompanied by abnormal regulation of the immune system, while many immune system disorders are influenced by the central nervous system (CNS) (Gibney and Drexhage, 2013; Kipnis, 2016; Dantzer, 2018; Reardon et al., 2018). The bi-directional ‘neuroimmune interface’ believed to underlie these interactions depends partly on mediators such as cytokines which, together with other neuroactive compounds including neurotransmitters and modulators, affect neuronal and glial function in the CNS. Conversely CNS neurons and glia, in addition to producing neurotransmitters and neuromodulators, generate some of the immune system mediators which regulate leukocyte function, establishing the potential for a two-way flow of information between the CNS and immune system. Components of the kynurenine pathway of tryptophan oxidation modulate neuronal excitability and neuro-glial plasticity in the CNS (Stone and Darlington, 2002; Badawy, 2017) but also regulate fundamental aspects of the immune system including inflammatory balance, immunosurveillance and tolerance (Belladonna et al., 2009; Mandi and Vecsei, 2012; Proietti et al., 2020; Gargaro et al., 2021).

Despite this overlap, neither the immune system mediators nor tryptophan metabolites alone provide a complete explanation for disorders which may involve both systems. It is therefore proposed that the two families of compounds function as an integrated inter-dependent network in which the broad changes in neural excitability and synaptic plasticity in the CNS, and the generalized control of immune system inflammatory balance and tolerance are modulated by kynurenines. This activity would determine the basal level of cell function across the neuroimmune divide. Cytokines and chemokines could then superimpose a layer of greater regional and functional refinement which generates the specificity required for organ and tissue selective bi-directional communication.

This perspective begins with a brief summary of kynurenine and cytokine biology, followed by a more detailed expansion of the integration concept with examples of interactions between cytokines, kynurenines, the CNS and immune system. The concept of ‘volume transmission’ in the CNS is discussed as the structural basis within which those molecular interactions can occur, with comments on the contribution of the blood-brain barrier (BBB) and glia. Finally, there is an exploration of the possible mechanisms by which the different actions of tryptophan metabolites and immune mediators can achieve the functional inter-dependence and tissue specificity for a neuroimmune interface with regional selectivity.

## Cytokines and kynurenines

### Immune system mediators and the central nervous system

Cytokines and chemokines are immune system mediators which define leukocyte phenotypes and regulate innate and adaptive immunity. They determine the balance of pro- and anti-inflammatory activity, and the susceptibility to autoimmune disorders, infection and malignancy. Importantly, the immune system mediators also have potent actions in the CNS originally recognized by their involvement in ‘sickness behavior’ which affects multiple affective and intellectual dimensions (mood, rationality, cognition) with symptoms of hyperthermia, apathy, reduced locomotion and socialization, and a centrally mediated loss of appetite, all indicating CNS involvement (Kent et al., 1992; Dantzer et al., 2008). Interleukin-1β (IL-1β) was considered primarily responsible for sickness behavior as its levels in the blood and brain correlated with symptoms in rodents and humans (Konsman et al., 2008; Harden et al., 2011; Felger and Miller, 2012), intracerebral administration reproduced the symptoms, and they were blocked by IL-1-receptor antagonist (IL-1ra) (Konsman et al., 2008; Huang et al., 2010). Subsequently it was realized that IL-1β has wide-ranging influences on neuronal excitability, synaptic plasticity, neurogenesis, cognitive function and neuro-degeneration – effects also prevented by IL-1ra or genetic deletion of *IL1B* (Koo and Duman, 2008; Spulber and Schultzberg, 2010; Clausen et al., 2011; Sugama et al., 2011; Haroon et al., 2012; Takemiya et al., 2017; Salvador et al., 2021). Antagonism of IL-1β also interfered with brain development (Spulber et al., 2011).

Other cytokines can affect the CNS (Felger and Miller, 2012; Galic et al., 2012; Haroon et al., 2012; Felger and Lotrich, 2013; Miller et al., 2013; Kipnis, 2016; Dantzer, 2018; Salvador et al., 2021), some examples of which are summarized in Table 1. Many, including IL-1β, IL-6 and Tumor Necrosis Factor (TNF) can be released by activated astrocytes or microglia (Heppner et al., 2015) as well as immune system cells, contributing to normal function and pathological CNS conditions (Deverman and Patterson, 2009). Increased levels of IL-1β in the basal ganglia and nucleus accumbens have been associated with modified behavioral control and affective state. They may contribute to anxiety, fear and depression in human subjects (Felger and Lotrich, 2013; Miller et al., 2013) and possibly underlie Major Depressive Disorder (MDD) or bipolar disorder (BD) (Harrison et al., 2009). Immune mediators may have special importance in neurodevelopmental disorders such as autism and schizophrenia (Deverman and Patterson, 2009), with lifelong effects on cell proliferation, migration, synaptic, and extrasynaptic junctions (Garay and McAllister, 2010; Martineau, 2013).

**TABLE 1:** Examples of the actions of cytokines on the central nervous system.

Name	Targets and actions on the CNS
IL-1β	- Mediates effects of stress on the CNS; releases neuroactive mediators from glia (T1, T2) - Modulates neuron activity with lasting changes in synaptic function (T3) - Promotes CNS development, growth, regeneration (blockade inhibits) (T4) - Contributes to neurotransmitter and cognitive dysfunction after injury (T5–T8) - Can promote neuronal apoptosis (T9) - Modifies glutamate receptor expression (T10–T13) - Polymorphisms may contribute to cognitive deficits (T14) - Interferes with trophic and stabilizing effects of growth including BDNF (T15–T18) - Stress increases IL-1β expression in hippocampus and enhances learning (T19–T21) - Modulates corticosterone production (T22) - Induces glioblastoma growth (T23)
IL-6	- Expressed in neurons and glia by LPS, IFN-γ or stress – generally inhibits neurons (T24–T26) - IL-6 can modulate synaptic transmission (T27, T28) - Activates glial metabolism and reduces neuronal metabolism (T29) - Regulates neuronal development, growth and regeneration (T30, T31) - Contributes to sickness behaviors and anxiety (T32) - Involved in schizophrenia, depression (T33) - Plasma and CSF levels increased by LPS, IFNs, IL1β (T34) - Induces changes in synaptic proteins contributing to ethanol dependence (T35, T36)
IL-10	- Promotes differentiation of anti-inflammatory cells; reduces pro-inflammatory cells (T37) - Inhibits neuronal differentiation (T38–T40) - Attenuates astroglial reactivity by (T41) - NMDAR induced release from lymphocytes (T42) - Released from glial cells and neurons, provides trophic support to neurons (T43) - Complex interactions and regulation of neurons and glia (T44, T45) - Blocks the inhibitory effect of IL-1β on long term potentiation (T46) -Facilitates LTP in hippocampus (T47) - Regulates pro-inflammatory cytokine generation and NO synthase in glia (T48, T49) - Induces haem oxygenase-1 (T50) - Prevents glutamate-induced death of cerebellar granule cells (T51) - Generally protective: reduces inflammatory cytokines; rescues neurons after ischemia (T52–T55) - Prevents hypoxic loss of AMPAR (T56) - Has reciprocal interactions with Nerve Growth Factor (NGF) (T57, T58). - Regulates GABA transmission and anxiety (T59) - Determines inflammatory status in MS, ALS (T44, T60) - Regulates myelination stability and inflammation (T61) - Upregulated by atypical antispsychotics (T62, T63)
IL-15	- Increased and released by local inflammation (T64, T65) - Promotes neurogenesis and maintains synaptic function especially GABA and 5-HT (T66, T67) - Increases in astrocytes after brain injury: contributes to damage (T68) **- I**nvolved in inflammatory reactions and microglial activation of MS, PD, AD (T69)
IL-16	- Inhibits sodium channels, AMPAR expression and glutamate transmission (T70) - Inhibit excitatory currents, calcium influx, and neurotoxicity (T71) - Reduces CNS neuronal damage (T70, T71)
IL-17	- Pro-inflammatory; enhances CCL2 and CXCL1 production and intercellular adhesion (T72) - Controls lymphocyte recruitment into CNS (T73) - Promotes neurodegeneration, damage and dysfunction (T74) - Promotes inflammation and cell loss after stroke (T75) - Contributes to progression of CNS inflammatory disorders (EAE, MS) (T76–T78) - May contribute to Parkinson’s disease (T79) - Raised levels in schizophrenia, with potential role in symptoms (T80) - Promotes autism-like phenotypes in mouse offspring (T81)
IL-18	- Pro-inflammatory: induces IFN-γ expression and potentiates IL-12 (T82) - Important mediator in many inflammatory conditions of CNS (T83) - Pleiotropic effects on cell communication (T84, T85) - Involved in the control of affective states and reward-related behavior (T86, T87) - Induction of IDO and quinolinic acid formation contributes to seizure susceptibility (T88) - Raised levels in schizophrenia (T89) - Produces neuronal and synaptic dysfunction: possible role in Alzheimer’s disease (T90) - Involved in neuroinflammation and degeneration? (T91) - Regulates hypothalamic function and stress reactivity (T92)
TNF	- Activates phagocytosis, proliferation, adhesion; promotes apoptosis of foreign cells (T93) - Modulates synaptic transmission and receptor localization; inhibits synaptic plasticity (T94) - Via TNFR1 enhances excitability by reducing GABA-A receptors (T95, T96) - Promotes AMPAR expression in cortex, increasing excitability (T54, T96) - May contribute to schizophrenia and bipolar depression (T95) - Increased in multiple sclerosis and models (T97)

References: T1: Koo and Duman, 2008; T2: Goshen et al., 2008; T3: Gajtko et al., 2020; T4: Spulber and Schultzberg, 2010; T5: Takemiya et al., 2017; T6: Clausen et al., 2011; T7: Sugama et al., 2011; T8: Spulber et al., 2011; T9: Liu et al., 2021; T10: Liu et al., 2013; T11: Viviani et al., 2006; T12: Zhu et al., 2006; T13: Lai et al., 2006; T14: Zhang et al., 2021; T15: Tong et al., 2012; T16: Patterson, 2015; T17: Rage et al., 2006; T18: Barrientos et al., 2004; T19: Jones et al., 2018; T20: Herrera et al., 2019; T21: Mai et al., 2019; T22: Fleshner et al., 1995; T23: Kai et al., 2021; T24: Harden et al., 2011; T25: Aniszewska et al., 2015; T26: Burton et al., 2011; T27: Gruol, 2015; T28: Sallmann et al., 2000; T29: Brown et al., 2014; T30: Yang et al., 2017; T31: Spooren et al., 2011; T32: Streit et al., 2000; T33: Schwieler et al., 2015; T34: Erta et al., 2012; T35: Gruol et al., 2020; T36: Hernandez et al., 2016; T37: Strle et al., 2001; T38: Perez-Asensio et al., 2013; T39: Sawada et al., 1999; T40: Laffer et al., 2019; T41: Balasingam and Yong, 1996; T42: Kvaratskhelia et al., 2009; T43: Zhou et al., 2009; T44: Burmeister and Marriott, 2018; T45: Lobo-Silva et al., 2016; T46: Kelly et al., 2001; T47: Nenov et al., 2019; T48: Ledeboer et al., 2000; T49: Molina-Holgado et al., 2001; T50: Lee and Chau, 2002; T51: Brooker and Mocchetti, 2001; T52: Thompson et al., 2013; T53: Oruckaptan et al., 2009; T54: He et al., 2017; T55: Kastin et al., 2003; T56: Savina et al., 2013; T57: Yanik et al., 2020; T58: Pietrzak et al., 2009; T59: Patel et al., 2021; T60: Porro et al., 2020; T61: Yang et al., 2009; T62: Sugino et al., 2009; T63: Kwilasz et al., 2015; T64: Maslinska, 2001; T65: Pan et al., 2013; T66: Gomez-Nicola et al., 2011; T67: Huang et al., 2009; T68: Li et al., 2017; T69: Rentzos and Rombos, 2012; T70: Hridi et al., 2019; T71: Skundric et al., 2015; T72: Hridi et al., 2021; T73: Wojkowska et al., 2017; T74: Swardfager et al., 2013: T75: Zhang et al., 2021; T76: Milovanovic et al., 2020; T77: Waisman et al., 2015; T78: Hong and Hutton, 2010; T79: Storelli et al., 2019; T80: Debnath and Berk, 2017; T81: Choi et al., 2016; T82: Gumusoglu et al., 2020; T83: Kaplanski, 2018; T84: Nakanishi, 2018; T85: Alboni et al., 2010; T86: Alboni et al., 2011; T87: Alboni et al., 2013; T88: Anderson and Rodriguez, 2011; T89: Syed et al., 2021; T90: Bossu et al., 2010; T91: Felderhoff-Mueser et al., 2005; T92: Sugama and Conti, 2008; T93: Pribiag and Stellwagen, 2013; T94: Rizzo et al., 2018; T95: Lee et al., 2019; T96: Lewitus et al., 2014; T97: Millett et al., 2020.

Chemokines (“chemotactic cytokines”) govern the movement and localization of cells (Ubogu et al., 2006; Lai and Mueller, 2021; Sawant et al., 2021) and are therefore crucial in the communication between cells of the immune system, neurons and glia. Fractalkine (CX3CL1) has received much interest as it is produced constitutively by neurons and restrains microglial activation. The deletion of fractalkine impairs CNS development and neurogenesis (Harrison et al., 1998; Cardona et al., 2006).

### The kynurenine pathway

**FIGURE 1:**
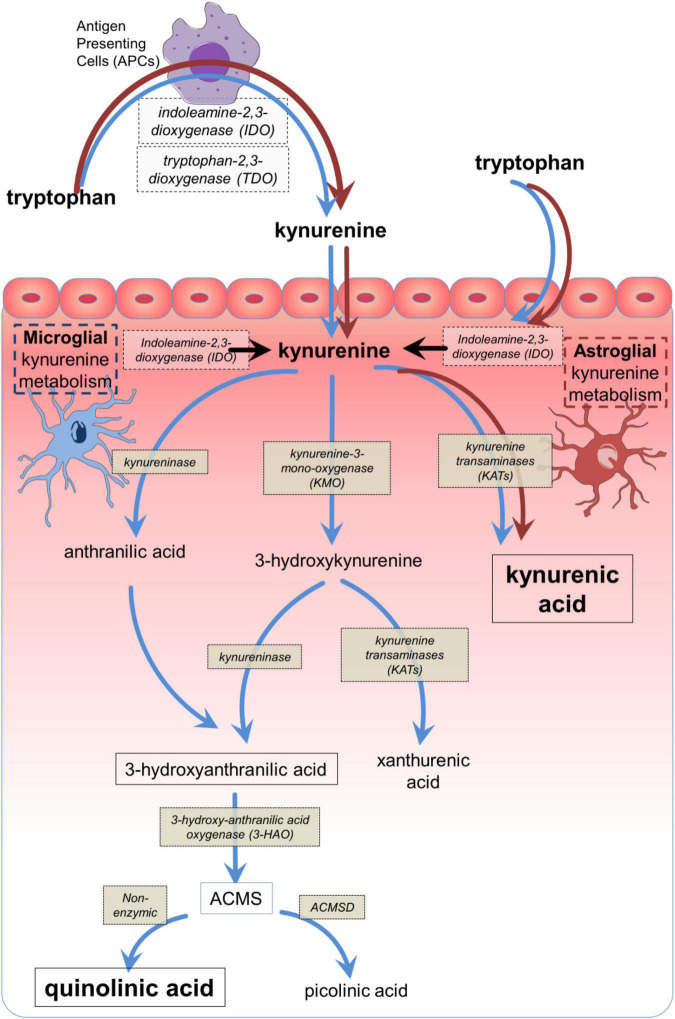
The main components of the kynurenine pathway. The main biologically active compounds of the kynurenine pathway, with their corresponding synthetic or catabolic enzymes from tryptophan to quinolinic acid or picolinic acid, depending on the presence or absence of ACMSD. The dark (brown) arrows indicate the dominant pathway which can be expressed by many cell types, especially antigen presenting cells including macrophages and their CNS counterparts, microglia. The light (blue) arrows indicate the abbreviated metabolism of tryptophan to kynurenic acid via kynurenine aminotransferases (KAT) which is expressed in astrocytes. The conversion of AA to 3HAA has been reported in mammalian tissues (Kotake et al., 1956; Kashiwamata et al., 1966; Baran and Schwarcz, 1990) but depends on the tissue and species (Saito et al., 1993; Fujigaki et al., 1998). ACMS, 2-amino-3-carboxy muconic acid semialdehyde; ACMSD, 2-amino-3-carboxy muconic acid semialdehyde decarboxylase.

**FIGURE 2:**
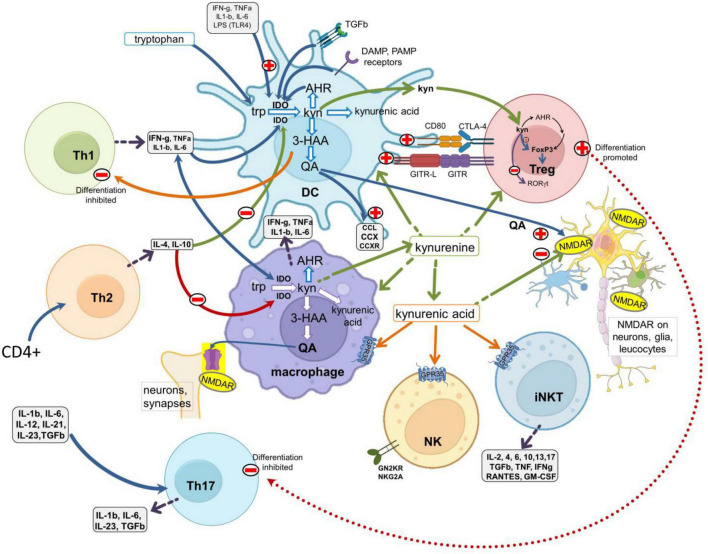
Interactions between the kynurenine pathway and the immune system. A schematic of major leukocyte populations showing the main sites of production and activity of the kynurenine pathway metabolites. The pathway is expressed constitutively in antigen presenting cells (DCs and macrophages) and is regulated by (a) ligation of the CD80/CD86 (B7) complex with CTLA-4 expressed by Treg cells; (b) the glucocorticoid-induced TNF receptor-related protein (GITR) and its ligand GITR-L; (c) the activation of receptors for inflammatory cytokines such as IFN-γ, IL-1β, IL-6, TNF, and with TLRs activated by Pathogen Associated Molecular Patterns (PAMPs, such as LPS, viral dsRNA and mammalian nucleotides) or Damage Associated Molecular Patterns (DAMPS). Activation of the kynurenine pathway generates 3HK, 3HAA, quinolinic acid and kynurenic acid. 3HAA inhibits Th1 cell activity, quinolinic acid is an agonist at glutamate (NMDA) receptors and kynurenic acid is an antagonist at glutamate receptors but can also activate GPR35 protein. NMDA receptors and GPR35 are expressed by many leukocytes and neurons in the CNS. Kynurenine produced by APCs acts as a paracrine agent to enter and influence lymphocytes, activating AHR to promote FoxP3 and Treg differentiation, but suppressing Th17 generation. TLRs, toll-like receptors; 3HAA, 3-hydroxyanthranilic acid; AHR, aryl hydrocarbon receptors.

The kynurenine pathway (Figure 1) accounts for over 90% of the metabolism of free tryptophan, which is oxidized initially to L-kynurenine primarily by indoleamine-2, 3-dioxygenase-1 (IDO1) (Stone, 1993; Stone and Darlington, 2002; Badawy, 2018; Platten et al., 2019). Interferon-γ was found to induce IDO1 expression in antigen presenting cells (APCs) (Yoshida et al., 1981; Yasui et al., 1986; Figure 2). This was considered to explain its inhibition of infection (Pfefferkorn, 1984) and the suppression of maternal lymphocyte attack on allogeneic embryos (Munn et al., 1998, 2002), partly by local tryptophan depletion and partly by the generation of downstream kynurenine metabolites. These observations introduced the concept of kynurenine metabolites as major factors in immunological function and tolerance.

Activation of the kynurenine pathway can be induced by molecular products of tissue injury or microbial fragments such as lipopolysaccharides (LPS), often acting via TLRs (Figure 2). These “sensory” routes complement the cytokine-kynurenine feedback cycles (Mezrich et al., 2010; Litzenburger et al., 2014; Li et al., 2016) which maintain kynurenine pathway activity beyond the early phases of induction in the phenomenon of ‘infectious tolerance’ (Grohmann et al., 2003; Andersson et al., 2008; Belladonna et al., 2009).

In the CNS, one product of the kynurenine pathway – quinolinic acid (Figures 1, 2) – is a selective agonist at glutamate receptors which respond to *N*-methyl-D-aspartate (NMDA) (Stone and Perkins, 1981; Perkins and Stone, 1983; Stone, 1993). The fundamental importance of NMDA receptors (NMDARs) lies in their contribution to excitatory synaptic transmission and the modulation of calcium fluxes and synaptic plasticity underlying learning and cognitive behavior (Traynelis et al., 2010; Stone and Darlington, 2013; Dubois and Liu, 2021; Kilonzo et al., 2021; McQuail et al., 2021; Schonewille et al., 2021). When over-activated by NMDA or quinolinic acid they can promote neuronal loss (Schwarcz et al., 1983; Stone et al., 2007; Guillemin, 2012; Stone and Darlington, 2013).

In contrast, kynurenic acid (Figures 1, 2) is an *antagonist* at all three glutamate receptor subtypes responding respectively to NMDA, α-amino-3-hydroxy-5-methyl-4-isoxazole-propionic acid (AMPA) or kainic acid (Perkins and Stone, 1982), but it is most potent at blocking postsynaptic NMDARs (Perkins and Stone, 1982, 1985; Ganong et al., 1983; Brooks et al., 1986; Ganong and Cotman, 1986) since it blocks the binding sites for glutamate and the co-agonist glycine (Ascher and Nowak, 1987; Birch et al., 1988; Danysz et al., 1989; Henderson et al., 1990). Glutamate antagonism accounts for the learning and cognitive deficits induced by kynurenic acid or procedures which increase its levels in the CNS (Stone and Darlington, 2002, 2013; Heisler and O’Connor, 2015). Increasing kynurenic acid levels before birth can lead to cognitive dysfunction in the offspring (Pocivavsek et al., 2019), corresponding to changes in CNS structure and synaptic function (Forrest et al., 2013a,b, 2015; Khalil et al., 2014; Pisar et al., 2014).

In addition to blocking these ionotropic glutamate receptors, cell activity may be affected by kynurenic acid activating the Aryl Hydrocarbon Receptor (AHR) (Opitz et al., 2011; Bessede et al., 2014) or the GPR35 protein (Wang et al., 2006; Berlinguer-Palmini et al., 2013; Alkondon et al., 2015; Mackenzie and Milligan, 2015; Resta et al., 2016), while paradoxical stimulant effects can arise indirectly by blocking the activation of inhibitory neurons. The overall effects of kynurenic acid therefore depend on the pattern of neuronal activity in the network of excitatory and inhibitory pathways (Stone, 2021). The balance between quinolinic acid and kynurenic acid influence neuronal plasticity, development, cognitive function, recovery after injury and specific aspects of behavior such as pain perception (Ciapala et al., 2021) and addiction (Morales-Puerto et al., 2021).

The kynurenine pathway is present constitutively in APCs such as macrophages and dendritic cells (Guillemin et al., 2001, 2005a; Jones et al., 2015). The interplay between these and other immune system cells determines the levels of innate and adaptive immune system functioning. Key intracellular enzymes of the kynurenine pathway are regulated by pro- and anti-inflammatory cytokines and feedback circuits such as those centered around the AHR (Belladonna et al., 2006; Opitz et al., 2011; Bessede et al., 2014; Litzenburger et al., 2014; Li et al., 2016).

#### The initial enzymes: IDO1, IDO2, TDO

Although the first enzyme of the kynurenine pathway – IDO1 – is induced primarily by IFN-γ (Yoshida et al., 1981; Yasui et al., 1986), activation is also produced or potentiated by type-1 interferons, IL-1β, IL-6, TNF, LPS and viral dsRNA (Hissong et al., 1995; Hissong and Carlin, 1997; Babcock and Carlin, 2000; Robinson et al., 2003, 2005; Dai and Zhu, 2010; Watts et al., 2011; Bessede et al., 2014; Murray et al., 2015; Yeung et al., 2015; Mondanelli et al., 2020). These factors usually activate IDO1 via Toll-Like Receptors (TLRs) in APCs. TLR9 activation by CpG oligodeoxynucleotides increases expression of the B7 (CD80/86) protein complex on DCs which induced IDO1 expression. IDO1 can use a wide range of indole-derived compounds as substrates, including melatonin and indolic amines generated by the intestinal microbiota (see Section “Dietary and microbial influences”).

Cytokines associated with anti-inflammatory resolution and tissue recovery, such as IL-4 and IL-10 inhibit IDO1 expression (Musso et al., 1994; Mueller and Schwarz, 2010), while IDO1 reduces IL-10 expression, creating a stable feedback level of IL-10. This is greatly reduced in IDO(–/–) mice but is restored in double knockouts of IDO1 and IL-10 (Metghalchi et al., 2015; Laumet et al., 2018).

IFN-γ also induces KMO, KAT and kynureninase in circulating monocytes (McIlroy et al., 2005; Varga et al., 2015) and DCs, even in the absence of IDO1 (Belladonna et al., 2006). The increased generation of 3-hydroxynurenine (3HK), 3-hydroxyanthranilic acid (3HAA) and quinolinic acid are important in the immune system and CNS as noted below.

Exposure to stress induces expression of cytokines and IDO1 (Lu et al., 2013; Tian et al., 2014; Lavieri et al., 2016; Pearson-Leary et al., 2016; Rowland et al., 2018; Johnson et al., 2019; Kim et al., 2019; Upthegrove and Khandaker, 2020; Lumertz et al., 2022). Mice lacking IDO1 or treated with the inhibitor 1-methyl-tryptophan do not show the loss of spontaneous mobility and social interaction which characterizes depression in the forced swim test (O’Connor et al., 2009a,b; Raison et al., 2010; Alexander et al., 2013; Lawson et al., 2013). IDO1 induction in the CNS contributes to depressive symptoms associated with pain (Alberati-Giani et al., 1996; Guillemin et al., 2003; Kim et al., 2012). Kynurenic acid may be a major factor in this, since its blockade of NMDARs can suppress chronic pain (Pineda-Farias et al., 2013; Vecsei et al., 2013).

IDO2 is less active than IDO1 but mediates the same metabolism of tryptophan to kynurenine (Fatokun et al., 2013) and may be important in the activity of IDO1 (Metz et al., 2014). IDO2 can be induced by activation of the AHR and its role in immunomodulation includes regulation of antibody production (Merlo et al., 2014). IDO2 exhibits some non-enzymic actions on targets such as GAPDH and Runx (Mondanelli et al., 2021; Merlo et al., 2022), some of which may not involve tryptophan oxidation (Mondanelli et al., 2021). Despite having a spectrum of cellular targets (Prendergast et al., 2014), deleting IDO2 does not produce any behavioral changes (Too et al., 2016).

Tryptophan is also metabolized to kynurenine by tryptophan-2,3-dioxygenase (TDO2) an enzyme with greater substrate selectivity for tryptophan. TDO is expressed mainly in the liver (Badawy, 2017, 2018), but is a key link between the external environment and the kynurenine pathway. Exposure to physical or mental stress induces activity in the hypothalamo-pituitary-adrenal (HPA) axis leading to an increased synthesis of adrenal gluco-corticosteroids. These hormones induce and activate hepatic TDO, contributing to the regulation of systemic kynurenine levels. Stress is also associated with heightened immune system activity in the brain, with social withdrawal and anxiety, increased neuronal activity and microglial activation (Yin et al., 2019). Cognitive and emotional changes may be induced, potentially leading to mental illness (de Kloet et al., 2005; Davidson and McEwen, 2012), even when the stress is encountered during gestation (Lupien et al., 2009). These negative effects of stress on cognition have been linked to the activation of tryptophan metabolism by TDO (Kiank et al., 2010; Miura et al., 2011).

## Limitations and uncertainties: Concentrations and correlations

Arguments about the roles of tryptophan catabolites and cytokines in the CNS often center around the relationship between their systemic and central concentrations. Several studies of blood and CSF cytokine concentrations have shown parallel qualitative and quantitative changes, including those on TGF-β (Liu et al., 2022) with one report of IL-6 levels in blood correlating highly with mood (Gadad et al., 2021). The CSF: serum ratio of IL-6 levels was sufficiently consistent to act as a potential biomarker of intracranial aneurysms (Kaminska et al., 2021), while increased levels of IL-6 in rat brain parenchyma occurred in parallel with plasma and CSF values and with the severity of traumatic brain injury (Chatzipanteli et al., 2012).

However, levels of cytokines in the tissues and organs can be highly variable. A cytokine panel used in patients with tuberculous meningitis indicated that all (except IL-10) were present in brain at levels lower than in CSF (Loxton et al., 2021), while a panel of 36 cytokines used by Lepennetier et al. (2019) exhibited little correlation between serum and CSF levels in a variety of neurological disorders. Similarly, measurements of TNF, IFN-γ, IL-6, IL-8, and IL-10 revealed no correlation between plasma and CSF levels (Ellison et al., 2005), and a meta-analysis of patients with MDD found no relationships between IL-6 or TNF levels in blood and CSF or with PET-scan markers of inflammation (Enache et al., 2019). A highly instructive study concluded that the levels of several cytokines were not correlated between serum and CSF following peripheral (knee) surgery and that the central levels exceeded those in the periphery (Bromander et al., 2012) leading to the conclusion that even a peripheral inflammatory stimulus could induce a substantial CNS inflammatory response.

In the case of kynurenines, many studies of depression have reported increased activity along the kynurenine pathway but this is not a universal observation (Quak et al., 2014; Dahl et al., 2015). There is a high variability which imposes limits on interpretation (Birnbaum et al., 2018; Ogyu et al., 2018; Birnbaum and Weinberger, 2020) and the weakness of correlations between compound concentrations and disease symptoms has been highlighted in meta-analyses. It has been suggested that this reflects variations in diagnostic criteria (Arnone et al., 2018; Hunt et al., 2020; Pedraz-Petrozzi et al., 2020; Serafini et al., 2020). The need for greater precision of diagnosis has been raised by Sublette et al. (2011) who reported changes in cytokine and kynurenine concentrations only in suicidally depressed patients, a finding consistent with other evidence (Brundin et al., 2017).

### From uncertainty to hypothesis

Overall, therefore, the data suggest that the CNS presence of complex molecules does not lend itself to meaningful correlations with symptoms or with the levels of other compounds – such as kynurenines – with very different permeabilities and kinetics. These difficulties may disappear under the present hypothesis, as discussed in depth below, since the essential correlations to examine are not between cytokines or between kynurenine metabolites, but are the cross-correlations between the two families of compounds, reflecting the complementary nature of their contributions. Such measurements of course still need the appropriate attention to diagnosis, cohort variations, and clearer definition of the CNS regions relevant to a particular disorder or symptom.

## An integrated kynurenine-cytokine system

Clearly, the cytokine and kynurenine families play fundamental and wide-ranging roles in the immune system and CNS, with confusion arising on how to relate either of these groups to disease symptoms or severity. The hypothesis proposed here is that the two sets of compounds exert complementary actions so that integration between them is required to explain aspects of disease. This idea will be expanded in the following sections by considering in more detail how that integration could arise, with an emphasis firstly on molecular interactions and then secondly on spatial factors. Subsequent sections will then discuss the structural and functional aspects of how and where these interactions can occur, focusing on volume transmission and the roles of glial cells and the BBB.

### Cytokine – Kynurenine interactions: Molecular aspects

**FIGURE 3:**
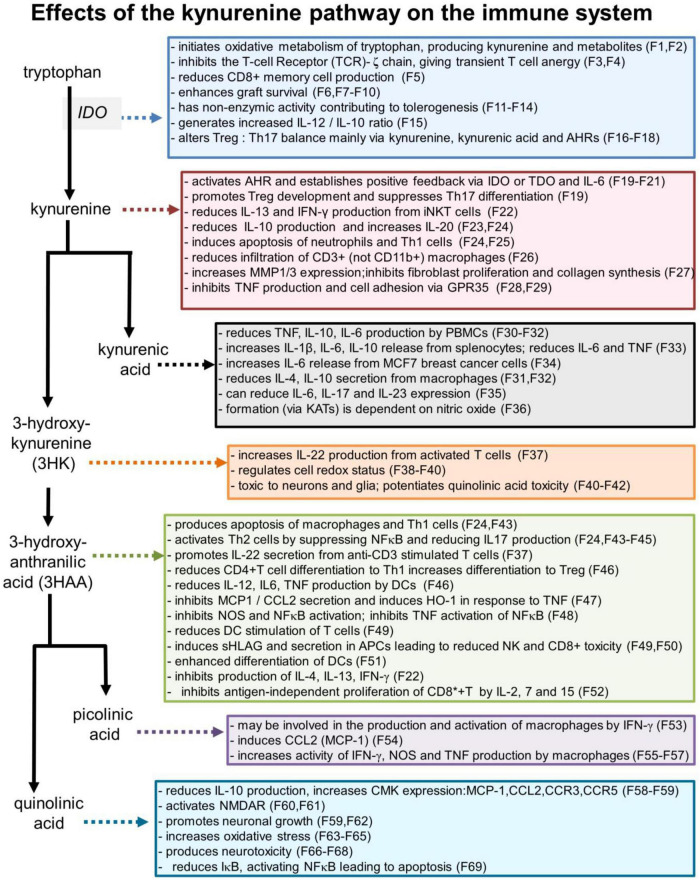
Selected effects of kynurenine pathway components on the immune system. Numbers refer to the corresponding references which are listed in a separate section of the references list, but which are summarized as follows: F1: Badawy, 2017; F2: Badawy, 2018; F3: Fallarino et al., 2006; F4: Eleftheriadis et al., 2016; F5: Kirillova et al., 2008; F6: He et al., 2015; F7: Zhang et al., 2018; F8: Huang et al., 2017; F9: Bock et al., 2013; F10: Xie et al., 2015; F11: Pallotta et al., 2011; F12: Belladonna et al., 2006; F13: Orabona et al., 2012; F14: Albini et al., 2017; F15: Juhl et al., 2020; F16: Favre et al., 2010; F17: Porro et al., 2020; F18: Baban et al., 2009; F19: Mezrich et al., 2010; F20: Litzenburger et al., 2014; F21: Li et al., 2016; F22: Molano et al., 2008; F23: Harden et al., 2016; F24: Fallarino et al., 2002; F25: El-Zaatari et al., 2014; P26: Elizei et al., 2015; F27: Poormasjedi-Meibod et al., 2016; F28: Barth et al., 2009; F29: Kolodziej et al., 2011; F30: Wang et al., 2006; F31: Metghalchi et al., 2015; F32: Fallarini et al., 2010; F33: Malaczewska et al., 2016; F34: DiNatale et al., 2010; F35: Elizei et al., 2017; F36: Luchowski and Urbanska, 2007; F37: Lowe et al., 2014; F38: Giles et al., 2003; F39: Goldstein et al., 2000; F40: Okuda et al., 1998; F41: Chiarugi et al., 2001; F42: Guidetti and Schwarcz, 1999; F43: Hayashi et al., 2007; F44: Terness et al., 2002; F45: Lee et al., 2010; F46: Lee et al., 2013; F47: Pae et al., 2006; F48: Sekkai et al., 1997; F49: Lopez et al., 2008; F50: Lopez et al., 2006; F51: Hill et al., 2007; F52: Weber et al., 2006; F53: Varesio et al., 1990; F54: Bosco et al., 2004; F55: Pastorino et al., 2004; F56: Ladomersky et al., 2018; F57: Melillo et al., 1993; F58: Croitoru-Lamoury et al., 2003; F59: Guillemin et al., 2003; F60: Stone, 1993; F61: Stone and Perkins, 1981; F62: Hernandez-Martinez et al., 2017; F63: Kubicova et al., 2013; F64: Santamaria et al., 2001; F65: Perez-De La Cruz et al., 2012; F66: Guillemin, 2012; F67: Schwarcz et al., 2010; F68: Bhat et al., 2020; F69: Qin et al., 2000.

Some of the more prominent interactions between cytokines or chemokines and the kynurenine metabolites of tryptophan are summarized in Table 1 and major actions of kynurenines on the immune system are shown in Figure 3.

Immune mediators can interact with glutamate-mediated transmission. TNF, for example, enhances release of glutamate from neurons and enhances its exocytotic release from glia (Santello et al., 2011; Santello and Volterra, 2012; Wang et al., 2017; Shim et al., 2018). Some of this release arises from glutaminase activity (Ye et al., 2013; Milewski et al., 2019) but glutamate uptake transporters are also inhibited (Olmos and Llado, 2014; Clark and Vissel, 2016).

Stimulation of NMDAR promotes TNF expression (McNearney et al., 2010) and TNF enhances NMDAR activity (Youn et al., 2008; Han and Whelan, 2010; Woods et al., 2021) including synaptic depolarization (Xu et al., 2006; Kawasaki et al., 2008; Li et al., 2009) mainly through TNFR1 receptors (Zhang et al., 2011; Del Rivero et al., 2019). This interaction extends to a regulation of NMDAR subunit composition (Weaver-Mikaere et al., 2013).

Interactions with AMPAR are less clear, since TNF modulates the expression and activity of AMPAR (Beattie et al., 2002; Stellwagen et al., 2005) and TNFR1 blockade reduces

AMPAR expression (Woolf and Salter, 2000; Beattie et al., 2002; Cingolani et al., 2008; Santello and Volterra, 2012). However, AMPAR-induced depolarization was not affected by TNF in other studies (Yang et al., 2002; Kawasaki et al., 2008; Xu et al., 2010). A negative feedback cycle, dependent on IDO1, stabilizes IL-10 expression (Metghalchi et al., 2015) thereby regulating AMPAR expression and glutamate-modulated neural excitability in the CNS (Savina et al., 2013).

Inflammatory mediators such as IL-6, IL-1β, and IFN-γ activate IDO1 and other kynurenine pathway enzymes primarily in APCs and CNS glia (Figure 2; Heyes et al., 1992a; Guillemin et al., 2001, 2005a; Andre et al., 2008; O’Connor et al., 2009b; Yamada et al., 2009; Jones et al., 2015; Murray et al., 2015; Yeung et al., 2015). Anti-inflammatory mediators such as IL-4 and IL-10 normally inhibit IDO1 expression (Musso et al., 1994). Since kynurenine enters and leaves cells by transmembrane diffusion and active transport (Belladonna et al., 2008; Sinclair et al., 2018) its production by APCs gives it a paracrine signaling role to cells which do not express IDO constitutively (Guillemin et al., 2005b; Andersson et al., 2008; Belladonna et al., 2008, 2009). This is crucial for immune function since kynurenine induces FoxP3 expression in CD4^+^ T cells, promoting differentiation to regulatory T cells (Tregs), but suppresses Retinoic Acid Receptor-related Orphan Receptor-γt (RORγt) which promotes differentiation to Th17 inflammatory cells (Figure 2). Dysregulation of the Treg: Th17 ratio has been associated with several autoimmune disorders (Favre et al., 2010; Jurado-Manzano et al., 2017) and may be dependent on epigenetic factors affecting IDO1 (Kawalkowska et al., 2019; Kmiolek et al., 2020; Huang et al., 2021). Treg control of the immune system is enhanced by their expression of CD39 and CD73 which hydrolyze ATP to adenosine, an important modulator of many neuronal activities (Stone et al., 2009). This nucleoside potently inhibits the release of synaptic neurotransmitters and other neuroactive compounds in the CNS and peripheral autonomic nerve terminals (Stone et al., 2007; Antonioli et al., 2013) contributing substantially to neuroimmune communication.

In addition to these direct actions of kynurenine and its metabolites, the localized consumption of tryptophan by IDO reduces the proportion of loaded tryptophanyl-tRNA, thereby activating General Controller Non-derepressible-2-kinase (GCN2k) and leading to reduced proliferation and increased apoptosis (Liu et al., 2014). Activation of GCN2k is synergistic with IL-12 and IL-6 in macrophages, leading to further IDO1 induction. The kynurenine generated will enhance Treg formation and produce an anti-inflammatory bias. However, kynurenine also promotes inflammasome activation in astrocytes, generating IL-1β (Zhang et al., 2020).

The effects of kynurenine are cell and context-dependent since it enhances IL-6 and IL-13 production by mast cells but inhibits IL-13 release from iNKT cells probably via AHR (Figure 2; Stone et al., 2007). This may be a factor linking kynurenine with obesity and autoimmune dysfunction (Nguyen et al., 2014; Stockinger et al., 2014; Moyer et al., 2016; Stone et al., 2018). Kynurenine inhibits expression of the Natural Killer (NK) cell activating lectin receptor NKG2, thus suppressing their anti-tumor properties. In human monocyte-derived cells, kynurenic acid reduces IL-6 and TNF release via AHR (DiNatale et al., 2010) and enhances IL-1β, IL-6 and TNF release, while suppressing IL-10 (Metghalchi et al., 2015).

An excellent example of the intimate functional relationship between immune mediators and kynurenine metabolites is the sickness behavior, described above. While early work implicated IL-1β, the behavior is also prevented by IDO1 inactivation (Jung et al., 2009; Gibney et al., 2013) emphasizing that downstream kynurenine metabolites make a significant to the cytokine-induced symptoms.

Changes of CNS function can be induced by depleting peripheral CD4^+^T cells (Bauer et al., 2007; Wolf et al., 2009; Kipnis et al., 2012). This leads to hippocampal neuronal loss and reduced cognition which is prevented by IL-4, indicating that neuronal viability and functional integrity are maintained by immune mediators secreted by peripheral lymphocytes (Kipnis et al., 2012; Kamimura and Murakami, 2019). Many such influences are probably mediated by the itinerant leukocyte populations passing through the CNS parenchyma.

#### 3-Hydroxyanthranilic acid and anthranilic acid

A key catabolite of kynurenine is 3-hydroxy-anthranilic acid (3HAA) (Figure 1). 3HAA entrains an anti-inflammatory cycle in which IFN-γ generated by antigenic activation of Th1 cells induces IDO1 in monocytes and APCs. The 3HAA generated by these cells then inhibits Th1 cells and promotes Th2 activity, thus reducing inflammatory activity (Fallarino et al., 2002). This will be enhanced by the paracrine transport of kynurenine into T cells (Sinclair et al., 2018), promoting Treg formation and further suppressing effector T cells. The result is an inhibition of pro-inflammatory cytokine release from Th1, CD8^+^, and NK cells (Figures 2, 3; Fallarino et al., 2002; Hayashi et al., 2007) while promoting anti-inflammatory Th2 activity (Fallarino et al., 2002, 2006; Terness et al., 2002; Darlington et al., 2010; Krause et al., 2011).

The ratio between anthranilic acid and 3HAA correlates with inflammatory status in many disorders (Forrest et al., 2006; Darlington et al., 2010; Heng et al., 2020; Huang et al., 2020; Jusof et al., 2022; Yan et al., 2022) and 3HAA contributes to allograft survival (He et al., 2015), synergistically with AHR activation (Gargaro et al., 2019). Kynurenine, kynurenic acid and 3HAA reduce NK cell activation and proliferation, potentially facilitating tumor development (Fallarino et al., 2002; Terness et al., 2002; Hayashi et al., 2007). The tolerogenic efficacy of 3HAA is partly achieved by promoting TGF-β generation and its maintenance of IDO^+^ CD8^+^ cells.

#### Quinolinic acid

**FIGURE 4:**
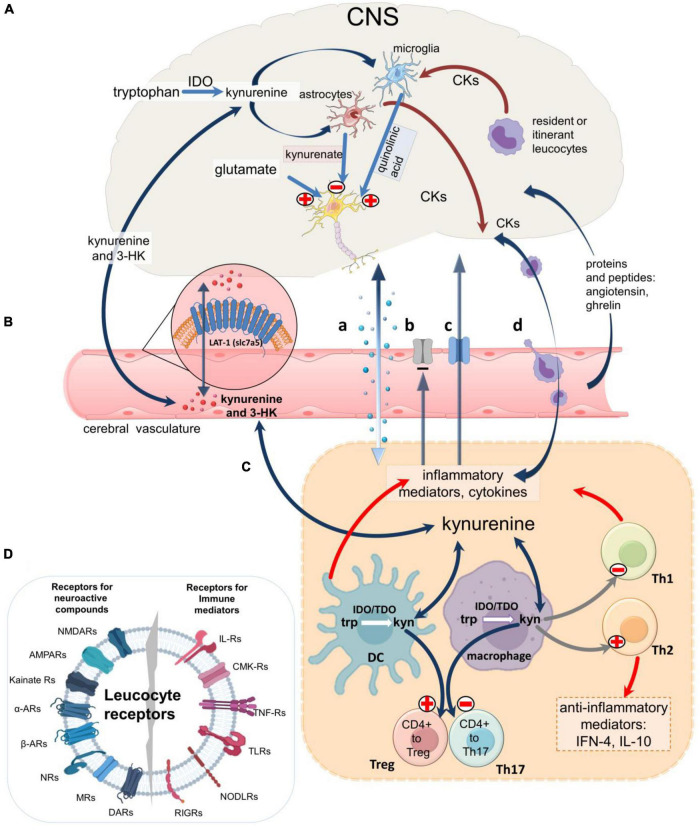
An overview of neuroimmune communication. Routes of communication between the CNS and immune systems including the blood-brain barrier. **(A)** IDO1 expressed in microglia produces kynurenic acid and quinolinic acid, a selective agonist on cellular NMDARs. Astrocytes produces only kynurenic acid which blocks receptors for endogenous glutamate on neurons, glia, and leukocytes, while glia and leukocytes resident in or passing through the CNS (‘itinerant’ cells) produce a variety of neuroactive cytokines and other inflammatory mediators which modulate glial activity and neuron excitability. **(B)** Kynurenine and 3HK generated in the CNS or in peripheral tissues cross the blood-brain barrier in both directions partly by diffusion and partly by the active Large Neutral Amino Acid Transporter (LAT-1). Larger immune mediator molecules gain access to the CNS by diffusion in areas of higher permeability (a), although some mediators may be excluded by persistent efflux transporters (b) and several have dedicated active transporters (c). Alternatively, immune mediators can be secreted by leukocytes which have crossed the cerebrovascular endothelium (d). **(C)** In all compartments the cytokines can regulate kynurenine pathway enzymes, partly via the balance of pro-inflammatory and anti-inflammatory interleukins. In the immune system kynurenine initiates a feedback generation of IDO and more kynurenine via Aryl Hydrocarbon Receptors. The kynurenine generated determines the expression of FoxP3 and RORγt and thus CD4 + differentiation to Treg or inflammatory T cells. **(D)** Leukocyte receptors for immune system mediators and neuroactive compounds produced by neurons and glia, emphasizing their susceptibility to ligands produced by cells of the immune system and the peripheral and central nervous systems.

Inflammatory activation of IDO in peripheral cells will increase plasma levels of kynurenine and 3HK which enter the CNS by diffusion and by the amino acid transporter LAT-1 (SLC7A5) (Sinclair et al., 2018; Figure 4). Kynurenine is then metabolized to kynurenic acid (in astrocytes) and quinolinic acid (in microglia and neurons) (Figure 1), (Guillemin et al., 2001; Sathyasaikumar et al., 2022) which leave the CNS slowly via the acidic amino acid transporter, blocked by probenecid (Figure 4). Stimulation of macrophages by IFN-γ therefore increases the synthesis and release of quinolinic acid (Heyes et al., 1992a; Chiarugi et al., 2000; Guillemin et al., 2005a; O’Connor et al., 2009a,b; Garrison et al., 2018) which tends to accumulate in the CNS. As an agonist at NMDARs (Stone and Perkins, 1981; Stone, 1993) quinolinate induces neuronal loss even at low concentrations if maintained for several days (Schwarcz et al., 1983; Whetsell and Schwarcz, 1989; Giulian et al., 1990; Guillemin, 2012) and has been linked with neurodegenerative disorders including Alzheimer’s disease (Majlath et al., 2014), Huntington’s disease (Schwarcz et al., 2010; Thevanavakkam et al., 2010), multiple sclerosis and amyotrophic lateral sclerosis (Guillemin et al., 2005b; Maya et al., 2018). High micromolar levels have been recorded in patients with immunodeficiency viruses, possibly contributing to the associated dementia (Heyes et al., 1992b; Kandanearatchi and Brew, 2012). It is likely that the pro-oxidant activity of quinolinic acid (Platenik et al., 2001; Santamaria et al., 2001; Seminotti et al., 2016; Hernandez-Martinez et al., 2017) and its subcellular targets leading to effects on apoptosis and autophagy (Silva-Islas et al., 2022) may also be relevant.

Importantly, quinolinic acid induces the expression of several key chemokines (including CCL2, CCL5, CXCL8, and CX3CL1) and chemokine receptors (CXCR4, CCR3, CCR5) (Guillemin et al., 2003). The resulting modulation of immune cell migration and mediator activity could in the CNS would exert a strong influence over neuronal function.

#### Kynurenic acid

In general, kynurenic acid is anti-inflammatory, suppressing TNF, IL-4 and IL-23 production by activated monocytes and reducing production of neutrophil peptides (Tiszlavicz et al., 2011). It also inhibits CD4^+^ cell differentiation to the Th17 phenotype via AHR (Wirthgen et al., 2018; Walczak et al., 2020a,b) and is an endogenous activator of GPR35 (Wang et al., 2006; Mackenzie and Milligan, 2015; Resta et al., 2016; Figure 2). This may carry novel implications for understanding the roles of kynurenic acid as new consequences of GPR35 activation are discovered such as those involving mitochondrial functioning (Wyant et al., 2022).

Kynurenic acid is released from neurons and glial cells (Figure 4; Turski et al., 1989; Swartz et al., 1990; Gobert et al., 2011; Heredi et al., 2019; Notarangelo et al., 2019). Activation of catecholamine β1- or β2-adrenoreceptors increased the levels of kynurenic acid *in vivo* or in brain slices or glial cultures (Luchowska et al., 2009), possibly accounting for some effects of catecholamines on the CNS and behavior.

Kynurenic acid blocks the ionotropic receptors for glutamate found on leukocytes – NMDA, AMPAR and kainate receptors (Lombardi et al., 2001; Boldyrev et al., 2004, 2012; Kvaratskhelia et al., 2009; Mashkina et al., 2010; Bhandage et al., 2017; Stone, 2020) (Figures 3, 4). The NMDA or AMPA receptors, and metabotropic glutamate receptors mGlu1R and mGlu5R (Pacheco et al., 2004) are analogous to those in the CNS and are associated with calcium influx and changes in cell proliferation, activation and differentiation (Lombardi et al., 2001; Nohara et al., 2015).

It is of interest that NMDAR activation induced the release of kynurenate *in vivo*, representing a further link between immune cell sensitivity and the CNS since glutamate generated by neuroglial activity will modulate itinerant leukocyte activity via their complement of glutamate receptors (Turski et al., 1989; Heredi et al., 2019). Glutamate induces release of anti-inflammatory IL-8, IL-10 and other cytokines from microglia and lymphocytes (Kvaratskhelia et al., 2009), so blockade of these receptors by kynurenic acid (Lombardi et al., 2001) may indirectly affect the release of several immune system mediators.

Modifying the endogenous levels of kynurenic acid for therapeutic purposes is a major and promising field of investigation (Stone, 2000a,b; Dounay et al., 2013, 2015; Kozak et al., 2014; Jayawickrama et al., 2015; Smith et al., 2016; Zadori et al., 2016; Jacobs et al., 2017; Majlath et al., 2018), especially as the neuroglial and leukocyte glutamate receptors are blocked by the same antagonists (Lombardi et al., 2001; Boldyrev et al., 2004; Kvaratskhelia et al., 2009; Mashkina et al., 2010; Bhandage et al., 2017) and respond similarly to changes in calcium availability (Nohara et al., 2015). Indeed, in isolated cell culture experiments, without the uptake and removal of kynurenate into cells or circulatory vessels, kynurenate is a highly potent antagonist at leukocyte NMDARs (IC50 of 400 nM) (Lombardi et al., 2001).

Both quinolinic acid and kynurenic acid have been implicated in Huntington’s disease (Forrest et al., 2010; Thevanavakkam et al., 2010; Stone et al., 2012), stroke (Darlington et al., 2007), multiple sclerosis (Sundaram et al., 2014; Boros et al., 2018; Platten et al., 2019), amyotrophic lateral sclerosis (Lee et al., 2017; Lovelace et al., 2017), schizophrenia (Wonodi et al., 2011; Stone and Darlington, 2013; Erhardt et al., 2017) and suicidality (Brundin et al., 2016, 2017) among other neurological and psychiatric disorders. Kindler et al. (2020) quantified kynurenine metabolites in blood and brain tissue of patients with schizophrenia. Several, including kynurenic acid, were increased in the prefrontal cortex, a region characterized by high levels of proinflammatory cytokines (Figure 4). The same population exhibited increased IDO1 activity (defined as the kynurenine: tryptophan ratio) in plasma, correlating inversely with patients’ attention. The results were consistent with elevated kynurenate concentrations causing abnormal mental behavior by blocking NMDA receptors. Quantification of kynurenine metabolites in the blood in MDD, BD and schizophrenia supported increased tryptophan metabolism, but with distinct metabolite profiles for the three disorders (Marx et al., 2021).

#### Picolinic and xanthurenic acids

Picolinic acid may have cell-protective and anabolic activity (Heng et al., 2016; Lovelace et al., 2017; Duque et al., 2020), with a possible pathological relevance in lupus nephritis for which it may be a suitable biomarker (Anekthanakul et al., 2021). Picolinic acid modulates macrophage metabolism and secretion of Macrophage Inflammatory Protein-1 (MIP-1) (Bosco et al., 2000; Rapisarda et al., 2002; Manzari et al., 2007).

Xanthurenic acid (Figure 2) can modulate neuronal activity and synaptic transmission in the CNS (Neale et al., 2013; Roussel et al., 2016; Sathyasaikumar et al., 2017). It appears to interact chiefly with metabotropic glutamate receptors and AHR but other targets may remain unidentified (Fazio et al., 2017). Xanthurenic acid has also been linked to the development of diabetes (Connick and Stone, 1985).

### Cytokine – Kynurenine interactions: Spatial aspects

The production of neuro-glial transmitters, neuromodulators, neurotrophins, cytokines and chemokines in the CNS yields a complex inter-cellular matrix (Section “Structural and functional factors underlying cytokine-kynurenine integration”) but some regional specificity is required to produce the varied, dynamic regulation of different behaviors, sensations (including pain), emotional reactions, and intellectual function, resulting from activity across the CNS and immune system. While most actions of kynurenine and its metabolites will be a generalized regulation of CNS development, excitability and plasticity, together with an organism-wide modulation of immune function, the present hypothesis argues that the reciprocal effects of immune system mediators on the CNS will produce a more localized, focused control of neuronal and glial activity. A mechanism is required to explain how such anatomical and functional complementarity could be generated between two such apparently complex and diffuse environments as the immune system and CNS.

Certainly one factor would be the distribution of receptors. Kynurenine pathway targets are widespread, including the glutamate receptors expressed by CNS neurons and glia, and many populations of leukocytes can be activated by quinolinic acid and blocked by kynurenic acid. Alternative targets for kynurenate such as AHR and GPR35 are present in a large fraction of cells, while effects on oxidative processes and redox status will affect almost all cell types. A highly specific distribution of receptors was first noted for IL-1 (Farrar et al., 1987) but has since been described for many others (Cayrol and Girard, 2022) with receptors located on different cells or groups (Gour and Wills-Karp, 2015; Hurdayal and Brombacher, 2017; Johnston and Bryce, 2017; Fairlie-Clarke et al., 2018; Younger et al., 2019; Wilson, 2021). In contrast, cytokines are often produced by distinct populations of cells, giving the compounds marked differences in kinetics and regional specificity (Scheller et al., 2021; Evavold and Kagan, 2022).

Viewed together, these various considerations contribute to the ability of individual cytokines to define specific aspects of CNS or immune system function superimposed on the more generalized regulation of cell behavior produced by the kynurenine pathway. This view could form the basis of selectivity mechanisms already recognized in peripheral tissues but now applied to the CNS, such as homing.

#### Homing

The term ‘homing’ refers to the directed migration of different cell types to appropriate locations affected by environmental or pathological changes. The concept is well established in peripheral tissues with the differential targeting of cells to various tumors or specific cell types within tumors. The homing factors are primarily cytokines and chemokines (Mikhak et al., 2006; Hanninen et al., 2011; Lupsa et al., 2018; Hoeppli et al., 2019; Xiong et al., 2020). The roles of CCR3 in prostate cancer (Guerard et al., 2021) and CXCR4 in leukemias (Burger and Buerkle, 2007) are good examples although many others including CXCR2 (Hoerning et al., 2011; Whilding et al., 2019) and the CCR2 driven homing of IL-23 expressing cells (Kara et al., 2015) have been well studied.

The spatial resolution of homing can be increased by the formation of complex molecular entities between classical cellular receptors and cytokine receptors. The dopamine receptor family, for example, has wide-ranging involvement in immune cell activity (Matt and Gaskill, 2020), extending to the formation of heteromeric complexes with other agents. The D5 dopamine receptors complex with chemokine receptor CCR9 (Osorio-Barrios et al., 2021) is an essential contributor to the homing of CD4^+^ T cells to the intestinal mucosa, and inhibiting its formation suppresses the phenomenon. The homing of CD4^+^ T cells is partly regulated by the interaction between the two T cell differentiating transcription factors FoxP3 and RORγt. The former complexes with AHR to enhance homing while RORγt inhibits homing (Xiong et al., 2020). As these are key targets of kynurenine and its downstream metabolites, this example emphasizes the importance of an integrated cytokine-kynurenine interface and its potential relevance to cellular and molecular complementarity. The value of targeting cytokines has been intensively explored for systemic autoimmune disorders such as arthritis (Malemud and Reddy, 2008; Hausmann, 2019; Kyttaris, 2019).

In the CNS, homing is likely to depend on the varied distributions of cytokines and chemokines localized to small regions of CNS and to specific cell types (Figure 4). One example is IL-1β which, although synthesized and released widely from neurons, astrocytes and microglia, acts on receptors that are restricted largely to granule cells of the dentate gyrus (Ban et al., 1993). This provides a mechanism by which changed levels of peripheral IL-1β may enter the CNS to produce effects that are spatially limited to those cells possessing receptors. The sensitivity of those receptors may be varied by up- or down-regulation within different, restricted areas of CNS, and the differential expression of receptor subunits will also present a different pharmacology (concentration dependence, sensitivity to antagonists etc.) which may be functionally relevant, especially if affected by disease.

Disorders of the CNS are already known in which cell homing may be a powerful factor. In multiple sclerosis, for example, the induced movement of inflammatory Th17 cells into early lesions is thought to be important in establishing myelitic plaque formation and development, and can determine the course of symptoms (Poeta et al., 2019).

#### Contact complementarity

The concept of homing was developed for cells attracted to a relatively homogeneous tissue or cell type but may not be adequate in the CNS. A more accurate view for neuroimmune communication might be based on ‘contact complementarity.’ This would involve a large number of molecular sites with which migrating cells make contact to interact and function in a complementary fashion. Those sites may encompass only a few – perhaps single – cells. Small groups of leukocytes would express a unique profile of ‘contact complementarity factors’ which interact with corresponding target molecules in the CNS. The most obvious such links would be the immune mediators and their receptors. Similarly, the chemical products of neurons or glia would act on leucocytic cytokine, chemokine, and neurotransmitter receptors (in addition to receptors on other neurons and glia).

Contact complementarity may resemble the spatial location of cells determined by their specific mix of proteins. In mouse brain, for example, microglia have high levels of CD11b, F4/80 and FcγRI in the hippocampus whereas in the frontal cortex CD11b is lower and CX3CR1 (fractalkine) is higher (de Haas et al., 2008). The difference emphasizes that complementarity could be based not only on the presence of a particular molecule, but also on the relative expression of several molecules.

The ability of the proposed mechanism to account for neuroimmune selectivity can be illustrated mathematically. If a group of leukocytes interacted with CNS cells based on the expression of a single molecule then, based on the existence of 100 relevant cytokines and related molecules influencing the CNS, there could be around 100 neuro-immunologically distinguishable regions of CNS. However, since the cell interactions could involve any number of mediators, in different combinations and at densities which may vary in time, there could be many millions of distinguishable CNS regions. This variety could generate localized activity affecting small groups of CNS neurons depending on the state of the cells concerned, local physiological inputs and the specific local extracellular chemical composition. It is this spectrum of contacts which could be prominently affected by the widespread changes in cell activity and polarization produced by environmentally modulated changes in IDO1 expression and its tryptophan metabolites.

Not surprisingly there will be exceptions to these rules, as some cytokines show a degree of non-selectivity for their receptors. Although some pairings are highly specific, as with the interaction between CXCL12 and its receptor CXCR4, some ligands can affect several receptor populations, and individual receptor subtypes may respond to multiple ligands (Deverman and Patterson, 2009). This implies that even at the ligand-receptor level, there is variability in the precise variety of receptors, and the relative concentrations of several ligands able to act on them. However, it is not the presence of any single ligand which will determine cell behavior, but the combined and relative presence of several.

Even the relatively few leukocytes within the CNS parenchyma or meninges would contribute to this regional complexity, since their products would access receptors on thousands of CNS cells (around 1 million per microliter). Complexity would be further enhanced by the diffusion and dilution gradients of compounds from several leukocytes.

In the CNS, the expression of complementarity factors will be influenced by the level of local neuronal activity (Figure 4). Similarly, glial polarization and activation, influenced by neuronal activity will alter the supply and removal of substrates and products. Changes in the kynurenine pathway (in neurons, astrocytes, glia or leukocytes), will produce corresponding effects on neuronal excitability. Leukocytes whose expression of complementarity factors or kynurenines has been modified by peripheral events will play a major part in these interactions, communicating and translating peripheral events in the immune system into neuronal modulation in the CNS.

The overall outcome of these interactions will be to generate a complex intercellular chemical matrix which would generate a different ‘map grid reference’ for each point within the CNS, rather like the point-to-point representations of the body surface in the sensory neocortex. The degree of commonality or overlap between cells in this chemical map will then (as for a zip or post code) determine the size and composition of each region.

#### Temporal aspects

There may also be a temporal aspect to cytokine-kynurenine interaction. Activation of IDO1 can occur over the first few hours of an appropriate stimulus, remaining elevated for 24 h or more, whereas IL-1β, IL-6 and TNF decline much more rapidly (Clanchy et al., 2022). Enzymes downstream of IDO1 (Figure 1) may become active in a similar time frame, even in cells lacking IDO (Belladonna et al., 2006).

In contrast, cytokines can exhibit a very wide range of time courses which are likely to amplify functional differences between the cytokines, as for IL-1 or IL-6 (Grellner, 2002; Uchida et al., 2007; Bai et al., 2008), IL-18 (Barrientos et al., 2009; Witt et al., 2011), IL-4 and IL-10 (van Meegeren et al., 2013), IL-15 (Shamsi et al., 2015), and IL-17 (Loof et al., 2016). Younger et al. (2019) summarize data showing that cytokines and their receptors are affected by injury on time scales of a few hours to several days, with similar conclusions after stress (Fatouros and Jamurtas, 2016). However, emphasizing the spectrum of cytokine activity, the administration of LPS to rats induced a peak of TNF and IL-1 expression in the plasma approximately 1 h later, while IL-1β, IL-1ra and IL-8 levels were detected 2–5 h after an allergy challenge in humans (Wagenmann et al., 2005). The use of fresh tissue may be more helpful, but the stimulation (by PMA) of fresh blood cells collected after burn injuries indicated peak levels of IL-6 or IL-8 several days later, a time frame far slower than kynurenine responses (Finnerty et al., 2006; Csontos et al., 2010). Cytokine induction after strokes can be similarly delayed (Nayak et al., 2012).

The existence of such a breadth in the temporal range of cytokine expression and activity would overlap that of the kynurenine pathway, such that the initiation, development or termination of cytokine activity could be modulated by the more generalized effects of kynurenine and its metabolites. This gives rise to the proposal that kynurenines provide a continually shifting ‘tonic’ baseline of cell activity, onto which the ‘phasic’ effects of immune system mediators are superimposed.

## Structural and functional factors underlying cytokine-kynurenine integration

If, as proposed here, the kynurenine pathway and cytokines function in a complementary fashion, partly accounting for the weak or conflicting correlations between tissue levels of either family of compounds in relation to disease symptoms or severity, how and where does this integration occur? This section addresses the question by considering the most important concept of ‘volume transmission,’ with additional comments on the contributions of glial cells and the BBB. Together, these features define the physical, structural factors involved in the neuroimmune interface.

### Mechanisms of integration: Volume transmission

The classical concept of synaptic transmission by fast neurotransmitters was that they acted at tightly apposed synaptic junctions, on a timescale of milliseconds, to modulate the polarization of neurons. Using the dominant amino acid glutamate as an example, fast transmission was mediated by AMPA and kainate receptors, with a slower excitation by NMDARs which generated much of the plasticity-based changes underlying behavior and cognition (Kandanearatchi and Brew, 2012; Dounay et al., 2013; Stone and Darlington, 2013; Varga et al., 2015; Kilonzo et al., 2021; McQuail et al., 2021). The subsequent activation of G-protein linked metabotropic glutamate receptors (Pacheco et al., 2004) extends the time frame of neuromodulation from seconds to minutes or hours.

**FIGURE 5:**
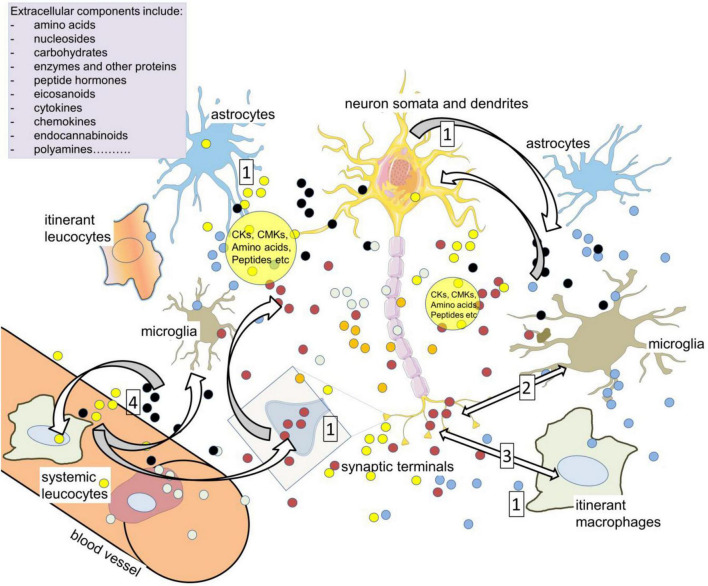
Volume transmission as a vehicle of neuroimmune communication. Compounds in the extracellular spaces of the CNS are released from neurons, glia, axons (including varicosities), cell bodies and dendrites in addition to synaptic terminals (1). Among the most active, relevant compounds are neurotransmitters such as glutamate, cytokines (CKs), and chemokines (CMKs), all of which can influence cell activity depending on the local expression of receptors and transporters, and all of which can act on – and be released by – resident and itinerant leukocytes. The large volume of brain tissue exposed to so many sources of active compounds led to the concept of ‘volume transmission.’ The primary neuroactive substances promote CK release (2) while CK receptors can release neurotransmitters and related compounds (3). The cytokines can also directly modulate neuron and synaptic function, affecting excitability and plasticity. The major neuroactive kynurenine metabolites include kynurenic acid (glutamate antagonist) and quinolinic acid (NMDAR agonist). The extracellular compounds may act on leukocytes in the CNS which later re-enter the circulation and interact with peripheral leukocytes (4). Kynurenine can enter and leave cells relatively readily, acting as a paracrine agent to maintain or enhance levels in nearby cells.

This view became modified by the recognition that slow neurotransmitters such as the monoamines were often released from axonal varicosities into a much larger volume of extracellular space, and this idea evolved further into the concept of “volume transmission” (Figure 5). The term encompasses the evidence that the release of most neuroactive compounds is not confined to the synaptic gap, but can arise from neuron terminals, somata, dendrites, axons, microglia, and astrocytes (Fuxe et al., 2007; Guidolin et al., 2007; Fuxe and Borroto-Escuela, 2016). This applies to fast transmitters as much as to peptides and proteins such as cytokines. Since receptors for many of the released compounds are also expressed on a broad range of cells (Christensen et al., 2016a,b; Figures 2, 3), their activation will depend on the overall quantity and time course of release from all these sources. These parameters will be highly variable since, for example, glutamate release is partly exocytotic from neurons and microglia and partly by efflux transporters, whereas astrocytes secrete glutamate in a more diffuse, continuous non-exocytotic manner (Malarkey and Parpura, 2008; Cresto et al., 2019; Mahmoud et al., 2019). The final concentration of any particular compound will therefore be a complex balance between synthesis, release and removal by transporters (Figure 5), with all these processes being subject to the rate and pattern of neuronal activity and the state of rest or activation of the glia (Acarin et al., 2000; Nimmerjahn et al., 2005; Paolicelli and Gross, 2011; Paolicelli et al., 2011; Erta et al., 2012; Trettel et al., 2020).

Since CNS cells secrete and respond to a variety of growth factors and cytokines, including IL-1β, IL-6, and TNF in addition to conventional neurotransmitters and neuromodulators (such as purines, peptides, endocannabinoids etc.), volume transmission will generate a highly complex medium in which active compounds can modulate the activity of neurons and glia with the appropriate receptors or targets at much greater distances than across synapses (Figure 3). The cell groups affected would be limited in size and localized to a restricted region of the CNS as determined by homing and complementarity factors, as discussed above. Theoretically, however, every cell in the CNS is exposed to a different extracellular medium composition, allowing almost a cell-to-cell communication between the CNS and immune system.

#### Neuroimmune transfer of information

Indeed, a central tenet of the neuroimmune concept is that leukocyte populations will participate in these chemical exchanges, responding to neuroglial secreted transmitters, modulators and cytokines, but also adding their various products of cytokines and proteins into the extracellular matrix. This will apply to resident and itinerant leukocytes in the CNS, so that the information on neuron and glial activity represented by the chemical composition of the CNS extracellular medium will be imprinted onto the cohort of leukocytes present in the CNS at that time. As those leukocytes eventually return to the systemic circulation, any subtle changes in phenotype will influence their interactions with other immune system cells, modifying their activity or differentiation and proliferation such that even a small number of cells ‘conditioned’ by the CNS extracellular medium could have a significant impact on immune system function.

Equally, of course, the molecular information in leukocytes entering the CNS will be determined by the state of the immune system in the previous minutes or hours, allowing the imprinting of that information onto neuron and glial activity.

It has been suggested that the level of local neural activity influences cytokine production and receptor expression which then generate a differential attraction of immune system cells into the CNS, further linking neural activity with neuroimmune communication (Kamimura and Murakami, 2019). Such a mechanism could well be a factor in refining these concepts.

#### Additional transfer mechanisms

In addition to the release and uptake of molecules by transporters or diffusion, cells may secrete high molecular weight compounds such as proteins and nucleic acids packaged into vesicles (exosomes) up to 100 nm diameter (Agnati and Fuxe, 2014). Transfer of the chemokine receptor and viral docking protein CCR5 provides an example of molecules which can be distributed to other cells in secreted exosomes (Camussi et al., 2011; Guo et al., 2018). Cells can also communicate by the formation of ‘nanotubes’ (Agnati et al., 2010; Agnati and Fuxe, 2014). These inter-cellular structures, approximately 200 nm in diameter, are up to ∼50 μm in length through which large molecules and complete intracellular vesicles can be transferred. They can be formed between different cell populations such as neurons and astrocytes, expanding the potential biological importance of moving high molecular components between phenotypically dissimilar pools. These processes may transfer key molecules from stressed or injured cells to healthy ones (Agnati and Fuxe, 2014). Most importantly for the present discussion is the possibility that they may allow the transfer of cell membrane molecules and receptors for neuromodulatory compounds (Bukoreshtliev et al., 2009; Agnati et al., 2010), creating a shifting pattern of cell phenotypes dependent on chemical influences and temporal changes.

### Mechanisms of integration: The blood-brain barrier

In addition to the complexity of volume transmission, the presence of a BBB will itself modify the movement of compounds to and from the CNS (Figure 4), contributing to the contradictory correlations noted above (section “Limitations and uncertainties: Concentrations and correlations”). Despite their protein nature and size (IL-1β is ∼17.5 kDa), many immune system mediators can cross the BBB structurally unchanged even in adult animals (Broadwell and Sofroniew, 1993; Capuron and Miller, 2011) reaching concentrations which can influence animal behavior (Banks et al., 2001, 2002). IL-1α, IL-1β, and IL-1ra have active transport into the CNS (Banks et al., 1989, 1994; Gutierrez et al., 1994). These are specific processes, as unrelated cytokines such as IL-2 were not transported (Waguespack et al., 1994). Conversely the active transport of IL-6 is not affected by IL-1 (Banks et al., 1994). Brain region-specific actions of some cytokines may result from regional variations in BBB function and selectivity (Moinuddin et al., 2000). In so far as neuroglial activity may influence specific transport processes such as these, it would contribute to general features of neuroimmune communication.

As with much larger proteins, cytokines (including chemokines) can also enter the CNS through leaky regions such as the circumventricular organs (CVO) (Konsman et al., 2008). Some, including IL-1β, act on cerebrovascular endothelial cells to stimulate the production of more IL-1β which is secreted into the brain parenchyma (Figure 4). As small amounts of IL-1β reach the parenchyma, they may activate glial cells to induce further IL-1β production and release into the extracellular space of the CNS, able to spread its influence by acting successively on other glia and neurons. Other proteins known to cross in CVO areas include IL-17A (Chen et al., 2022), angiotensin (McKinley and Oldfield, 1998), and ghrelin (Uriarte et al., 2021).

Some smaller compounds ‘leak’ across the capillary endothelial layer outside the CVO to reach biologically active concentrations within the CNS. A classical example is morphine which crosses the barrier poorly but still sufficiently to induce a rapid and effective analgesia (Banks et al., 1995). Some compounds bypass the barrier by transcellular processes involving movement by cytoskeletal networks, while some are moved within intracellular vesicles which afford protection from modification. Molecules moving in this way include large molecular weight proteins such as insulin and transferrin and smaller but critically important trophic peptides such as NGF, BDNF (Crowe and Morgan, 1992; Pan et al., 1998a,b) the glycoprotein erythropoietin and related compounds (Banks et al., 2004).

#### Leukocytes

Intact leukocytes can cross or by-pass the BBB giving them, and the cytokines they express, direct access to the CNS parenchyma. In particular, elements of later stage, adaptive immune activity including T cells are able to access the CNS via diapedesis across the choroid plexus and by the binding between chemokine CCL20 and its chemoattractant receptor CCR6 (Axtell and Steinman, 2009). This system is particularly important in the access of Treg and Th17 cells and their penetration into regions of the limbic system (Sallusto et al., 2012). Here again IL-1β is involved as it acts on its receptor on endothelial, ependymal and astrocytic cells to induce the expression of CCL2 and promote cell migration. Indeed, IL-1β is often viewed as a ‘Trojan horse’ of the immune system as it increases the overall permeability of the BBB (Shaftel et al., 2007), facilitating the ingress of other cytokines and intact leukocytes (Figure 4). TNF plays a significant role in the attraction of monocytes into the CNS (D’Mello et al., 2009).

Leukocytes also gain access from the vascular circulation to the arachnoid mater, from where they can enter the CSF and the brain parenchyma through the meninges (Schlager et al., 2016). Among the leukocytes with this ability are several functionally distinct subsets of CD4^+^ T-cells, with evidence that marked changes of learning are associated with neuronal loss in the hippocampus following the depletion of peripheral CD4^+^ T cells, implying that the presence of these cells makes a significant contribution to neuronal and synaptic plasticity (Wolf et al., 2009). It is also claimed that populations of CD4^+^ T lymphocytes are partly responsible for neuronal damage (Liblau et al., 2013) although this can also be caused by CD8^+^ T cells penetrating the CNS and releasing destructive enzymes and cytokines (Medana et al., 2000; Huse et al., 2008; Liblau et al., 2013).

### Mechanisms of integration: Glial cells

Microglia are derived embryologically from monocytic myeloid cells and phenotypically they resemble systemic macrophages (Ginhoux et al., 2010). They produce a variety of cytokines including IL-6, IL-1β, and TNF, and chemokines such as CCL1, CCL5, CCL12, some of which induce the migration of systemic immune system cells (Hanisch, 2002; Kettenmann et al., 2010; Kraft and Harry, 2011). They may also produce eicosanoids, growth factors, purines, neuropeptides, polyamines, proteases and other compounds which might fulfill niche roles in specific aspects of immuno-neural communication (Sternberg, 2006; Hanisch and Kettenmann, 2007; Muzio et al., 2007; Fabry et al., 2008; Ekdahl et al., 2009; Talbot et al., 2016; Pavlov and Tracey, 2017; Kanashiro et al., 2020; Scheiblich et al., 2020). Microglia also identify and destroy aberrant and non-self cells, often presenting fragments as antigens (Miyanishi et al., 2021).

Once systemic monocytes have crossed into the CNS, the mediators they release activate resident microglia to become motile and to secrete mediators which modify neuronal and synaptic function (Ransohoff and Perry, 2009; Spulber and Schultzberg, 2010; Garay et al., 2013; Giovanoli et al., 2016). The activation of TLR4 on microglia induces their transformation from a stabilizing M2 to a reactive M1 phenotype (Rodriguez-Gomez et al., 2020). This may be crucial in the ability of microglia to influence neuronal degeneration, regeneration and neurogenesis in the aftermath of CNS injury or infection (Leiter et al., 2016; Diaz-Aparicio et al., 2020).

It has been suggested that a population of immune system-derived ‘extra-neural’ cells exists in the meninges and choroid tissue. While their relationship to other cell types is not clear, they may respond to foreign molecules and regulate central neuroglial activity by secreting their own spectrum of compounds (Becher et al., 2006; Tambuyzer et al., 2009), generating a ‘microglia-cytokine axis’ (Werneburg et al., 2017). These cells may play a specialized role in regulating axo-dendritic development and synapse formation, affecting network development and neuronal plasticity (Paolicelli and Gross, 2011; Paolicelli et al., 2011).

#### Receptors

Integration of the CNS and immune systems exists even at the level of individual receptors. The synaptic regions of neurons have a concentrated expression of IL-1 receptor (IL-1R) which can occur physically associated with the GluN2B subunit of NMDARs (Gardoni et al., 2011). Both IL-1β and NMDA can modify the expression of these complexes and their precise localization within the synapse. Immune system mediators secreted by microglia can modulate synaptic neurotransmission by altering the ratio of glutamate expression and sensitivity to NMDA, AMPA or kainate (Bauer et al., 2007; Bessis et al., 2007; Masgrau et al., 2017). Indeed, lymphocyte-generated cytokines can induce the expression, in microglia, of receptors and transporters for glutamate GABA, acetylcholine and catecholamines (Lisak et al., 2009), providing an almost direct ability of cytokines to interact with these and modulate neuronal firing and plasticity. These molecular interactions can be bi-directional: IFN-γ potentiates GABA-mediated neuronal inhibition (Flood et al., 2019) while GABA inhibits microglial activation (Bjurstom et al., 2008).

A novel perspective on the neuroimmune relationship has been gained from interfering with the D3 dopamine receptor, which is localized exclusively to CNS microglia. Blockade or deletion of D3 caused activation of those glia, with symptoms of depression and increased levels of IL-1β, IL-6, and TNF in dopaminergic neurons (Wang et al., 2020; Bassett et al., 2021).

### Neuronal influences on immune system cells

The discussion of immune system effects on the CNS is probably easier to conceptualize than the converse interaction, so it is important to note experimental examples showing how CNS activity directly influences the immune system.

The reciprocal production of secreted neuro-glial transmitters (Biber et al., 2007), neuromodulators and cytokines is reflected in the existence of receptors for glutamate on glial cells and leukocytes, illustrated by the glutamate-induced release of IL-8 and IL-10 from lymphocytes (Kvaratskhelia et al., 2009). The release of IL-10 from microglia is potentiated by presynaptic NMDAR activity leading to increased postsynaptic activation of non-NMDA (AMPA and kainate) receptors (Paetau et al., 2017; Stone, 2021). Kynurenic acid blocks many of these receptors, indirectly affecting mediator release.

Intracerebral injections of NMDA increased the number of NK cells in the systemic circulation, with increased levels of TNF. These responses were reduced by mild stress acting on CNS efferent glutamate-mediated (NMDAR-activated) pathways demonstrating their ability to influence systemic immunological function (Podlacha et al., 2016).

Mild stress also increased CNS microglial activation and cytokine expression (Ben-Shaanan et al., 2016; Jones et al., 2018; Du Preez et al., 2021). However, the cytokine changes were detected in both the CNS microglia and in systemic macrophages (Dantzer, 2006) but were not the basolateral amygdala or perirhinal cortex (Jones et al., 2018). The changes occurred in parallel with improved learning and so were not dependent on cytokine movements from periphery to CNS. They were presumably induced by stress-induced neuroglial activation in the hippocampal region from where they reached the systemic circulation.

Efferent neuroimmune communication is clear in the stimulation of limbic dopaminergic reward neurons of the Ventral Tegmental Area, which activates the peripheral immune system (Luscher and Malenka, 2011; Matt and Gaskill, 2020). Dopamine is important in movement and locomotion, and in the regulation of affective states and psychotic disorders. Microglial dopamine receptors (Luscher and Malenka, 2011; Matt and Gaskill, 2020) mediate structural and biochemical changes. Most lymphocyte populations release dopamine (Fan et al., 2018; Talhada et al., 2018) and express dopamine receptors (Cosentino et al., 2007; Huck et al., 2015; Levite, 2016; Talhada et al., 2018) although there are differences between T cell subsets, and between resting and activated cells (Besser et al., 2005; Watanabe et al., 2006). Leukocyte dopamine receptors, and those on CNS and systemic macrophages, affect cytokine production, cell adhesion properties, and mobility (Kirillova et al., 2008; Mignini et al., 2013; Coley et al., 2015; Nolan et al., 2019). The Th17 population of CD4^+^T cells is involved in the dopaminergic regulation of multiple sclerosis, arthritis, inflammatory bowel disease and Parkinson’s disease (Melnikov et al., 2018, 2020; Capellino, 2020), supporting the profound mutual influence of the immune and nervous systems.

## Dietary and microbial influences

The potential role of intestinal microbiota in modulating the host neuroimmune systems has been the subject of several reviews (Anderson et al., 2019; Cryan et al., 2019; Berding et al., 2021; Spichak et al., 2021). Tryptophan is not synthesized by mammalian tissues and is mainly obtained from dietary sources. In contrast, bacteria synthesize tryptophan in the shikimate pathway via anthranilic acid and, while the tryptophan is then available for protein synthesis, bacteria also catabolize it along the kynurenine pathway. Since tryptophan, kynurenine and 3HK are transported readily across the intestinal epithelium, their movements will affect levels of kynurenine and its metabolites in the bacteria and host. Changes in diet, disease or drugs affecting the intestinal microbiome will then have a significant impact on host neural, immune and behavioral factors. This view may be related to the concept that anthranilic acid levels seem to reflect the presence of infection or inflammation (Darlington et al., 2010; Badawy, 2018).

The recent discovery that the bacterial anthranilic acid derivative *Pseudomonas* Quorum Sensor (PQS; 2-heptyl-3-hydroxy-4-quinolone) modulates cytokine release and IDO expression in murine and human cells is an important extension to this concept. It reveals that a microbially important molecule, derived from the metabolism of tryptophan and which is not produced by mammals, can have highly significant effects on the host immune system (Ogbechi et al., 2022). The implication would be that changes in bacterial or host tryptophan metabolism could have reciprocal effects on both organisms as an example of ‘inter-kingdom signaling’ (Pacheco and Sperandio, 2009) between prokaryotes and eukaryotes.

Since IDO1 substrates include several indole derivatives produced by bacteria, the kynurenine pathway may represent a critical evolutionary development to detoxify and destroy chemicals not produced by the host organism. This would be consistent with the expression of AHR and their activation by kynurenine or kynurenic acid leading to the activation of catabolic cytochrome oxidases as well as the expression of transcription factors such as FoxP3 and RORγt which define the differentiation of Treg and Th17 cells respectively (Omenetti and Pizarro, 2015; Morris et al., 2017; Su et al., 2022). The Th17 cells concentrated in the intestinal walls and Peyer’s patches could exert a profound influence on host immunity (Bhaumik and Basu, 2017; Cheng et al., 2019; Chen and Tang, 2021), modulated by dietary and microbial tryptophan and anthranilic acid. Interestingly, one of the most potent known activators of AHR is FICZ, an endogenous metabolite produced by the photo-oxidation of tryptophan.

## Implications for drug development

To date, much drug development research has concentrated on individual compounds or families and specific molecular targets but this may lead to, at best, treatments for small cohorts of patients or, at worst, failure to achieve significant results across a varied, inadequately characterized population. If major examples of molecular interaction, such as has been attempted here, prove to be valid then a combined screening of relevant compounds in different families might be more fruitful, leading to therapeutic agents or combination thereof, which are more beneficial for more patients.

In the case of cytokines and kynurenines recent results lend support to this view with evidence that cytokines and kynurenine metabolites overlap in their relevance to clinical disorders. Comai et al. (2022) have noted that the respective contributions of these molecular groups may contribute to the differential etiology of MDD and BD. This is consistent with current clinical views on these conditions, but the introduction of a molecular basis for their differences could lead to an increasingly refined diagnosis and selection of patients for drug trials. That, in turn, could lead to drugs which are much more disease and symptom-specific.

Reinforcing this argument, few cytokine-related drug candidates have reached clinical trials, or those that have done so have proved insufficiently active or selective (Poeta et al., 2019; Lai and Mueller, 2021). The difficulty may lie in the potency of many cytokines coupled with the need to target a limited number of immune system cells or a restricted region of CNS. Reaching a sufficient concentration of drug within a limited volume of tissue will almost certainly be associated with unwanted effects elsewhere, especially given the relative non-selectivity of some cytokine receptors (Deverman and Patterson, 2009).

The kynurenine pathway is also the subject of much activity in drug discovery, with several clinical trials in progress (Pires et al., 2022). Autoimmune disorders represent a major focus for kynurenine-related drug development (Baumgartner et al., 2019; Huang et al., 2020, 2021; Ogbechi et al., 2020; Krupa and Kowalska, 2021) together with cancer (Prendergast et al., 2017, 2018; Muller et al., 2019; Opitz et al., 2020; Walczak et al., 2020a,b) and other conditions (Dounay et al., 2013, 2015; Kozak et al., 2014; Jayawickrama et al., 2015; Smith et al., 2016; Zadori et al., 2016; Jacobs et al., 2017).

In the CNS, both quinolinic acid and kynurenic acid have been implicated in Huntington’s disease (Stone, 1993, 2000a,b; Stone and Darlington, 2002; Thevanavakkam et al., 2010), stroke (Darlington et al., 2007), multiple sclerosis and related conditions (Sundaram et al., 2014; Lee et al., 2017; Lovelace et al., 2017; Boros et al., 2018; Majlath et al., 2018; Platten et al., 2019), schizophrenia (Erhardt et al., 2017), MDD, suicidality (Brundin et al., 2016, 2017) and other disorders.

For more than 50 years it has been thought that some depressive disorders are caused by lowered levels of 5HT in the CNS, supported by the apparent therapeutic success of Selective Serotonin Reuptake Inhibitors (SSRIs), even though counseling can be more effective (Mackay et al., 2009). However, the serotonin hypothesis has been brought into serious doubt by Moncrieff et al. (2022) who concluded that there is a lack of firm supporting that view. This may make it more likely that the kynurenine pathway has a more important role in depressive disorders and may be the target of future antidepressant therapies. Broadening the understanding of kynurenine pathway activity and interactions across the neuroimmune interface may promote this change of perspective to the benefit of many patients with a variety of complex disorders.

## Summary and conclusion

The concept of a neuroimmune interface attempts to explain how systemic insults can affect brain function and how aspects of behavior affect the immune system. The kynurenine pathway is viewed as pivotal in these interactions, mediating the translation of CNS neural activity to changes in the surveillance, defensive and tolerance activities of the immune system. Conversely, the latent presence of the kynurenine pathway in cells of the immune system, under the control of cytokines and chemokines, enables immune system activity to influence the CNS. Similarly, activity in peripheral immune cells, with the immunologically active and neuroactive factors they produce represents an additional force modulating neuronal and glial functions, contributing to subtle changes in a variety of behaviors. Overall, the low intensity modulation of neuronal excitability and synaptic plasticity by glutamate, NMDARs and the kynurenine pathway, it is proposed, lays the shifting foundation of a ‘tonic’ modulation of neuronal and glial activity upon which the ‘phasic’ effects of immune system modulators are superimposed at a localized, perhaps even cellular, level. Efforts to explain clinical disorders and to develop new diagnostic tools or treatments should take into account both the immune system mediators and kynurenine pathway metabolites for a more complete explanation and understanding of the neuroimmune and immuno-neural interface. The potential for producing highly localized changes in selected CNS regions could improve the safety, tolerability and efficacy of drugs for a range of psychiatric and neurological disorders as well as autoimmune conditions.

## Author contributions

TS wrote the initial draft. FC, Y-SH, N-YC, LD, RW, and TS contributed to the subsequent discussions and modifications. All authors contributed to the article and approved the submitted version.

## Abbreviations

3HK: 3-hydroxy-kynurenine
3HAA: 3-hydroxyanthranilic acid
AHR: aryl hydrocarbon receptor
BDNF: brain-derived neurotrophic factor
CNS: central nervous system
CSF: cerebrospinal fluid
GM-CSF: granulocyte-macrophage colony-stimulating factor
IDO-1: indoleamine 2, 3-dioxygenase-1
IDO2: indoleamine 2, 3-dioxygenase 2
KAT: kynurenine aminotransferase
KMO: kynurenine monooxygenase; kynurenine-3-hydroxylase
NMDA: *N*–methyl–D–aspartate
TDO2: tryptophan 2,3-dioxygenase
TLR: toll-like receptor.
